# Carcinoembryonic antigen and cytokeratin 20 in peritoneal cells of cancer patients: are we aware of what we are detecting by mRNA examination?

**DOI:** 10.1038/sj.bjc.6604189

**Published:** 2008-01-15

**Authors:** M Kowalewska, M Chechlinska, R Nowak

**Affiliations:** 1 Department of Molecular Biology, The Maria Skłodowska-Curie Memorial Cancer Centre and Institute of Oncology, Roentgena 5, Warsaw 02-781, Poland; 2 Department of Immunology, The Maria Skłodowska-Curie Memorial Cancer Centre and Institute of Oncology, Roentgena 5, Warsaw 02-781, Poland

**Sir**,

In a recently published paper in your journal, Katsuragi *et al* (2007) show that peritoneal recurrence in patients with early-stage gastric cancer relates to the levels of carcinoembryonic antigen (CEA) and/or cytokeratin 20 (CK 20) transcripts in the peritoneal lavage specimens from these patients. The authors are convinced that a positive RT-PCR result identifies the presence of ‘micrometastatic cells’. In our opinion, there are no grounds for this presumption. We and others have raised the issue of the nonspecific expression of so-called tumour markers by activated lymphoid cells (Kowalewska *et al*, 2006). CEA and CK 20 transcripts are markers of limited value for the detection of cancer cells for several reasons. Both markers have been shown to be expressed by the haematopoietic cells of healthy volunteers and of patients with chronic inflammatory diseases (Jung *et al*, 1998, 1999; Champelovier *et al*, 1999, Vlems *et al*, 2002). The qRT-PCR-based techniques of CEA and CK 20 detection present the same limitations as conventional RT-PCR, as researchers were unable to set the cut-off values to distinguish between cancer and haematopoietic cell expression (Bustin *et al*, 2004; Schuster *et al*, 2004). Especially in the experimental setting studied by Katsuragi *et al* (2007), one should consider that CEA expression may be induced in peripheral blood mononuclear cells by cytokines (Jung *et al*, 1998; Goeminne *et al*, 1999), as increased concentrations of an array of cytokines characterise peritoneal fluids of cancer patients (Chechlinska *et al*, 2007). Furthermore, peritoneal lavage samples of cancer-free patients were found to contain CEA transcripts (Broll *et al*, 2001). In addition, normal granulocytes that are likely to be recruited to peritoneal cavity (Hau, 1990) have been shown to express CK 20 (Jung *et al*, 1999; Kruger *et al*, 2001). In fact, the increased numbers of granulocytes as well as of activated lymphocytes were demonstrated in the peritoneal fluids of gastric and colon cancer patients as compared with those of cancer-free controls (Olszewski *et al*, 2007).

Unfortunately, none of these limitations, although widely discussed in numerous papers, have been considered or even discussed by Katsuragi *et al* (2007). In effect, the observed correlation between the positive CEA and CK 20 qRT-PCR signals and cancer recurrence seems reasonably well documented, whereas the statement that the authors detect ‘free cancer cells’ in their study, expressed already in the title and then subsequently used through the methods section to discussion, is totally unwarranted.

Considering the above, the measurements of the markers described by Katsuragi *et al* (2007) may reflect the presence of inflammatory cells rather than micrometastatic cells: the more so because as many as 35% of cytology-negative/PCR-positive patients developed no peritoneal metastases. Nevertheless, prognosis for PCR-positive patients was shown to be significantly worse than for PCR-negative patients, and this was in accordance with the recent data of Crumley *et al* (2006) and Deans *et al* (2007), who linked inflammation symptoms with adverse prognosis in patients with gastric cancer. However, there are simpler and cheaper methods than qRT-PCR to assess inflammatory parameters.

The study of Katsuragi *et al* (2007) is also methodologically flawed. The *GAPDH* gene was used as an internal control and a reference gene. The *GAPDH* gene is known for the presence of its numerous pseudogenes, which makes it inadequate as a reference gene in non-DNased total RNA samples, such as those prepared by Katsuragi *et al* (2007). In addition, the reader learns nothing about the standardisation of RNA quantities subjected to reverse transcription, necessary to make a reliable comparison of samples containing different cell counts.

Finally, Katsuragi *et al* (2007) use the inexplicable and inappropriate term ‘tumour nucleotides’, and for reasons that are far from obvious the disseminated cancer cells are called ‘isolated’ cells, although no isolation or enrichment procedure was performed. The authors have not applied the idea of micrometastases detection in cancer cell-enriched populations, one of many detection methods focused to enhance specificity and the only one which produced an assay that received FDA clearance (Smerage and Hayes, 2006).
